# Lip and oral lesions in children with Down syndrome. A controlled study

**DOI:** 10.4317/jced.52283

**Published:** 2015-04-01

**Authors:** Sadeq-Ali Al-Maweri, Bassel Tarakji, Ghadah A. Al-Sufyani, Hashem M. Al-Shamiri, Giath Gazal

**Affiliations:** 1 Assistant Professor, Department of Oral and Maxillofacial Sciences, Al-Farabi Colleges of Dentistry and Nursing, Saudi Arabia; Department of Oral Medicine and Diagnosis, Sana’a University, Yemen; 2 Professor, Head Department of Oral and Maxillofacial Sciences, Al-Farabi Colleges of Dentistry and Nursing,, Saudi Arabia; 3 Department of Dental Surgery, Al-Kuwait Teaching Hospital, Sana’a, Yemen; 4 Lecturer, Department of Oral and Maxillofacial Sciences, Al-Farabi Colleges of Dentistry and Nursing; 5 Assistant Professor, Department of Oral Maxillofacial Sciences, College of Dentistry, Taibah University

## Abstract

**Background:**

Down syndrome (DS) is the most common chromosomal abnormality affecting numerous organs, including the orofacial region. The objective of the present study was to assess the prevalence of lip and oral soft tissue lesions, with particular emphasize on the incidence of fissured tongue, lip fissures and angular cheilitis, among individuals with DS in Yemen.

**Material and Methods:**

This controlled cross-sectional study included 50 children with DS (6-18 years), and 50 age-and gender-matched healthy controls. The prevalence of orofacial soft tissue lesions was evaluated in both groups. Data were analyzed by Chi-square and Fisher tests, and p < 0.05 was considered to be statistically significant.

**Results:**

Ten orolabial lesions were identified among the subjects. The most frequently seen lesions were: Fissured tongue (78.0%), lip fissures (64.0%), angular cheilitis (38.0%) and Cheilitis (14.0%). The frequencies of these lesions were significantly higher in children with DS than healthy controls (P< 0.001). Most of lip fissures were in the lower lip, and 80% of the fissures were in the midline.

**Conclusions:**

The prevalence of lip and oral lesions among individuals with DS is remarkably high. Hence, oral physicians should be more aware of the orofacial findings seen more frequently in this genetic disorder.

** Key words:**Down syndrome, lesions, lips, oral.

## Introduction

Down syndrome (DS), or trisomy 21, represents the most common chromosomal abnormality associated with intellectual impairment (1). One-third of DS individuals are severely mentally challenged and in the past 5-10% were institutionalized (2). Clinically, DS is characterized by generalized hypotonia, neurological changes, structural cardiopathy, respiratory problems and a greater risk of infection, increased risk of leukaemai, dental anomalies, and orofacial dysmorphology (1,3,4).

The most common orofacial findings in DS include mouth breathing, open bite, relatively enlarged and protruding tongue, drooling, fissured tongue, malocclusion, low level of dental caries and poor oral hygiene (1,2,4-6).

Fissured tongue is the most common oral soft tissue finding in DS, with a recorded prevalence between 10% and 95% (7-9). It appears that fissured tongue is more frequent in individuals with DS than the general population, and its frequency rises with increasing age (10).

The incidence of angular cheilitis also appears high in individuals with DS (2,10,11), and has been attributed to depressed nasal bridge and muscular hypotonia which cause an open mouth and protruding tongue (11). Lip fissures, sometimes termed cracked or fissured lips, are also commonly seen in DS individuals, compared with other patients with learning disability as well as compared with the general population (2).

Numerous reports have documented the prevalence of dental caries and periodontal diseases in individuals with DS worldwide (1,3-6). However, the literature is scarce regarding lip and oral lesions in individuals with DS. We could find only 5 studies documenting orolabial findings (fissured tongue, fissured lips, angular cheilitis) in the English literature (2,8,9,11,12), and none has been written regarding the same in Yemen and neighboring Arab countries. Therefore, the present study aimed to assess the prevalence of orolabial lesions and conditions, with particular emphasis on the incidence of fissured tongue, angular cheilitis and lip fissures, among a group of Yemeni patients with DS, as compared to healthy controls.

## Patient and Methods

This controlled cross-sectional study, conducted between May and July 2014 in Sana’a, Yemen, included 50 children with DS and 50 age- and gender-matched healthy schoolchildren as controls. Children with DS were recruited from Al-Eyman institute for individuals with special needs, the largest special needs school for the handicapped in the city. All the children had been previously examined and diagnosed medically as DS patients according to the institute’s medical records. All children with DS lived at home (i.e. none were institutionalised). The inclusion criteria implemented were: (1) cytogenetic diagnosis of trisomy 21, (2) adequate cooperation from the children, and (3) obtainment consent from the children’s parents. The exclusion criteria were presence of detrimental systemic diseases, compound disability, and extremely uncooperative children. The healthy controls were randomly selected from one public school located in the same neighbourhood.

The study was approved by the Research and Ethics Committee, Faculty of Medicine and Health Sciences, Sana’a University, Yemen. Informed consent was obtained from the parents and the school authorities before the subjects were included in the study.

Demographic data such as, age, gender and residence were recorded for each child. Clinical examination was conducted for both groups by a single oral medicine specialist (Al-Maweri SA) under a strict protocol with the help of a standard registration form. Extra and intra oral examination was performed using electrical overhead light, mouth mirror, tweezers, gauze and wooden tongue depressor. Oral lesions were evaluated using standard international diagnostic criteria (13). The diagnosis of lip fissures and angular cheilitis was based on the clinical criteria described by Scully *et al.* (2). Presence and location of lesions were recorded accordingly. Children with any type of lesions were referred to the department of Oral Medicine, Faculty of Dentistry, Sana’a University, for appropriate treatment.

-Statistical analysis:

SPSS version 20.0 was used for data entry and analysis. Descriptive statistics were obtained, including percentages and frequencies for categorical data and means and standard deviations for numerical data. Chi-square test was used to compare proportion of lip and oral lesions between the groups. A significance level of *P* < 0.05 was considered.

## Results

Majority of the subjects in both groups were males (62%). The mean age of the DS children was 12.66 years (range: 6-18) with 42% were in the 10-13 year age group ( Table 1).

**Table 1:**
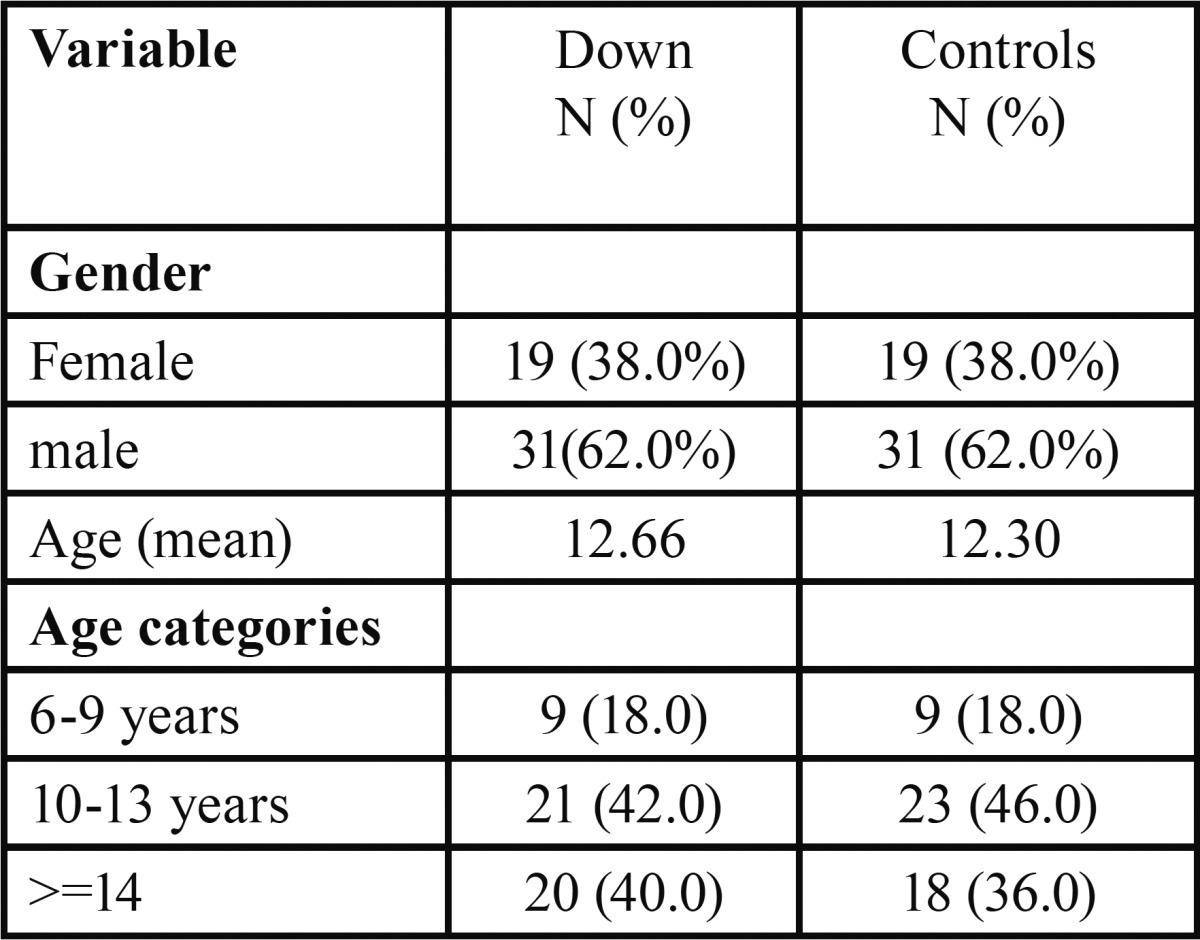
Demographic data of the study groups.

 Table 2 presents distribution of lip and oral soft tissue lesions in both groups. Ten lesions were identified. The most frequently observed orolabial lesions among DS children were fissured tongue (78%), lip fissures (64%) and angular cheilitis (38%). Children with DS had significantly higher proportion of fissured tongue, fissured lip, angular cheilitis, gingival hyperplasia and cheilitis, as compared to healthy controls (*P* < 0.001).

**Table 2:**
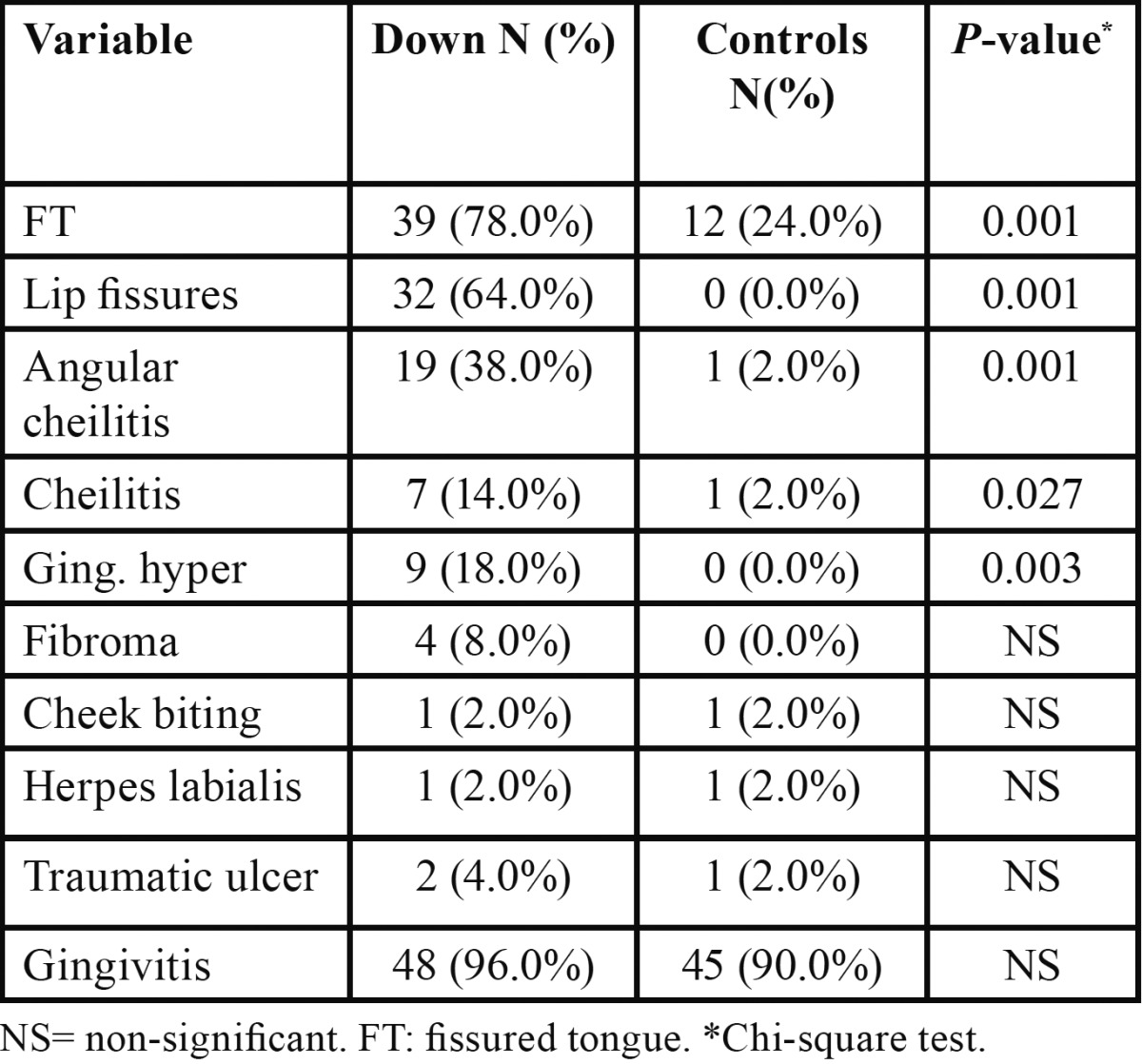
Proportion of orolabial lesions in both groups N (%).

Gender-wise comparison showed that lip fissures were more common in females than males (73.7 % vs. 58.1%) whereas fissured tongue was observed more frequently in males (83.9% vs. 68.4%); however these differences were not statistically significant. Angular cheilitis was observed with the same prevalence among males and females ( Table 3).

**Table 3:**
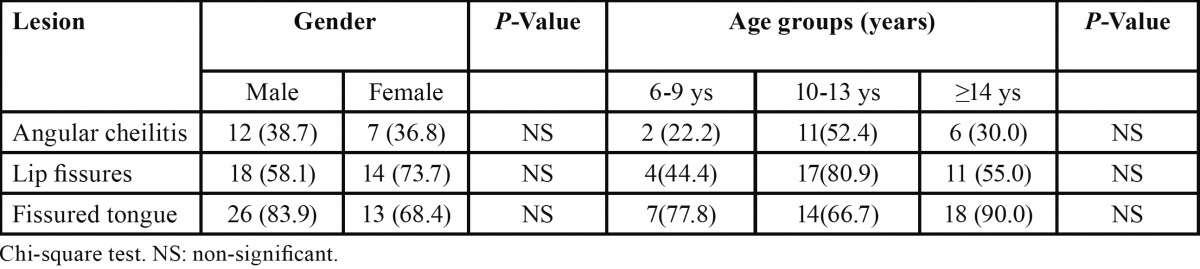
Distribution of the most frequent lesions (angular cheilitis, lip Fissures and fissured tongue) among children with Down syndrome according to gender and age groups N (%).

The results showed slight non-significant differences in the prevalence of fissured tongue, lip fissures and angular cheilitis between the age groups. The young age group showed the lowest presentation of these lesions ( Table 3).

**Figure 1:**
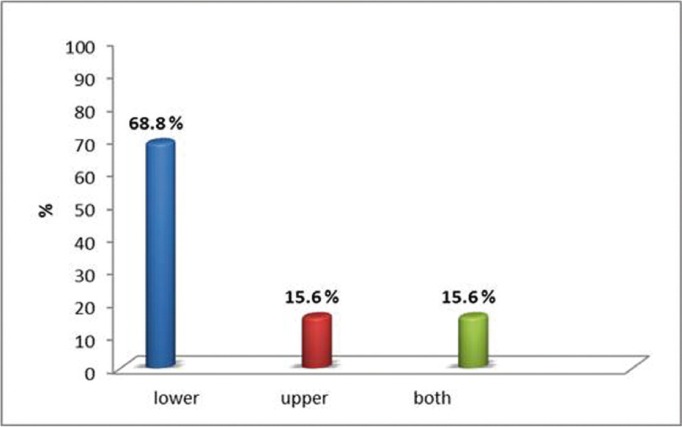
Distribution of lip fissures in upper and lower lips.

Majority (68.6%) of lip fissures were seen in lower lip, 15.6% in upper lip and 15.6% in both lips. The most common site of such fissures was in the midline (Fig. 1).

There was no correlation between the presence of different types of lip lesions, cheilitis, fissures and angular cheilitis (*P*> 0.5).

## Discussion

Information of the patterns of development of disease in a population is important because it can act as a database for the planning of public oral health policies. Hence this study was carried out to investigate the incidence of the various lip and oral lesions associated with DS. To the best of our knowledge this is the first study undertaken to assess these lesions among DS subjects in Yemen, and is one of the very few studies worldwide that investigated orolabial lesions among DS subjects utilizing control subjects.

In the present study, ten orolabial lesions were identified among the study subjects. The most common prevalent lesions were fissured tongue, angular cheilitis, lip fissures and cheilitis.

Fissured tongue prevalence was significantly higher in DS subjects than in control subjects. This finding is consistent with other studies, which reported a high prevalence of fissured tongue in patients with DS (8,9,11). The frequency of fissured tongue in the general population varies from 0.6% to 30.6% (14-16). However, the reported prevalence of this condition among DS individuals is between 20.0% and 95.0% (7-9,11). The rate of fissured tongue in our study (78.0%) coincides with these findings. Moreover, in agreement with previous studies, fissured tongue was seen more frequently in males and older age groups (9-11). Ercis *et al.* (10) suggested that tongue fissuring is associated with DS and that its rate may increase parallel by the age of the patient.

Lip fissuring was a common finding in DS subjects (64.0%), as compared to 0.0% in control group. This finding is very similar to the prevalence rate among Spanish DS patients (12,17). However, this rate is much higher than the 27.0% prevalence rate reported by Scully *et al.* (2). In our study, the midline of lower lip was the main location for lip fissures. This finding is in line with findings reported by other investigators (2). The etiology of lip fissures is still unknown. One theory suggested that in general population a congenital decrease in the size and number of mucous glands in the lip might predispose lip fissures but there is no evidence for this theory in DS (18). Other suggested causes of lip fissuring in general population include mouth breathing, avitmainosis, outdoor occupation, smoking and bacterial and fungal infection (19). Mouth breathing is a common finding in DS subjects owing to lip incompetence, a protruding tongue, and drooling and frequent rhinitis caused by a narrow air passage. Scully *et al.* (2) indicated that lip fissures were related to Candida albicans, mandibular prognathism and lip eversions. Further, Butterworth *et al.* (12) attributed lip fissuring to trauma and low grade infection.

Angular cheilitis was the third most common lesion among DS subjects, affecting more than one third of the sample, with equal distribution among males and females. The frequency of angular cheilitis was also significantly higher in DS subjects than controls (38.0% vs. 2.0%). This result corroborates previous findings by other investigators elsewhere (2,11). Angular cheilitis is an acute or chronic inflammation of the skin and contiguous labial mucous membrane at the angles of the mouth (20). Predisposing factors for angular cheilitis include mechanical, infective, nutritional, or immune defects. The high incidence of angular cheilitis in DS subjects seen in this study may be caused by Candida albicans as a result of drooling and immune defects in these children (2). It has been reported that patients with DS have a tendency for orofacial infections, especially with candida species (21). In 2002 study, Scully and colleagues investigated the association of lip lesions with Candida albicans in a group of DS patients. The authors found Candida was cultured from the patients with DS, who had angular cheilitis twice as frequently as in those with no lip lesions. However, the study could not identify weather Candida was a cause or effect of these lesions (2).

Cheilitis was found in 14.0% of DS individuals as compared to only 2.0% of healthy controls, which is consistent with earlier reports (9,11). Ercis *et al.* (10) attributed such lesion to depressed nasal bridge and muscular hypotonia, causing an open mouth and protruding tongue. Persistent mouth opening due to relatively large tongue in a reduced oral cavity may lead to mouth breat-hing, drooling and chapped and cracked lower lip.

Gingival hyperplasia and generalized gingivitis were also dominant in DS subjects. It is likely that poor oral hygiene coupled with systemic and local factors account for greater frequency of gingival diseases among this group of people. These findings support previous findings (4,6,22,23).

## Limitation and Conclusions

This study has several limitations. The main limitation of the present study is the relatively small sample size. Therefore, it was difficult to establish gender- and age-related trends. Additionally, generalization of the results must be made carefully as this study sample might not reflect the DS population in Yemen. Another limitation is the lack of microbial and/or serological investigations (due to financial restrictions). Such investigations could have explained and shed some light on the etiological factors of these lesions in DS subjects. Nevertheless, despite these limitations, the results of this survey should be useful for providing more information about orofacial lesions among subjects with DS. In turn, dentists and physicians should be aware of the diversity of orofacial anomalies present in this underprivileged group of the community.
